# Gamification of Learning Deactivates the Default Mode Network

**DOI:** 10.3389/fpsyg.2015.01891

**Published:** 2016-01-07

**Authors:** Paul A. Howard-Jones, Tim Jay, Alice Mason, Harvey Jones

**Affiliations:** ^1^Graduate School of Education, University of BristolBristol, UK; ^2^Sheffield Institute of Education, Sheffield Hallam UniversitySheffield, UK; ^3^School of Experimental Psychology, University of BristolBristol, UK

**Keywords:** default mode network, working memory, memory, reward

## Abstract

We hypothesized that embedding educational learning in a game would improve learning outcomes, with increased engagement and recruitment of cognitive resources evidenced by increased activation of working memory network (WMN) and deactivation of default mode network (DMN) regions. In an fMRI study, we compared activity during periods of learning in three conditions that were increasingly game-like: Study-only (when periods of learning were followed by an exemplar question together with its correct answer), Self-quizzing (when periods of learning were followed by a multiple choice question in return for a fixed number of points) and Game-based (when, following each period of learning, participants competed with a peer to answer the question for escalating, uncertain rewards). DMN hubs deactivated as conditions became more game-like, alongside greater self-reported engagement and, in the Game-based condition, higher learning scores. These changes did not occur with any detectable increase in WMN activity. Additionally, ventral striatal activation was associated with responding to questions and receiving positive question feedback. Results support the significance of DMN deactivation for educational learning, and are aligned with recent evidence suggesting DMN and WMN activity may not always be anti-correlated.

## Introduction

Games offer incentivised conditions that are remarkably effective in engaging players in goal-directed behavior (Przybylski et al., 2010). This ability of games to engage their players has prompted the idea that “gamifying” learning experiences (i.e., embedding the learning in a game-like context) might improve learning outcomes. This interest may reflect common-sense reasoning that, if gamification leads to a more rewarding environment for learning, we might be more engaged and so learn more rapidly. A scientific basis for this idea is provided by data showing incentives can enhance a range of cognitive processes (Krawczyk and D’Esposito, 2013) including working memory, which is considered a strong predictor of educational learning (Gathercole et al., 2004; Alloway and Alloway, 2010). Additionally, human fMRI studies have shown reward can increase activity in prefrontal and parietal regions associated with working memory (Pochon et al., 2002; Taylor et al., 2004; Krawczyk et al., 2007; Beck et al., 2010; Savine et al., 2010). In a verbal working memory task, for example, incentive motivation can modulate performance with amplification of activity within prefrontal and visual association regions selective to processing the perceptual inputs of the stimuli to be remembered (Gilbert and Fiez, 2004).

Efforts to localize working memory function during learning have converged on a dorsal fronto-parietal network that is activated in demanding tasks that require the processing of material presented (Corbetta and Shulman, 2002). Activation of this network has been recorded in efforts to learn a range of new abilities that include complex mathematics, second language acquisition (Lopez-Barroso et al., 2015) spatial skills (Nemmi et al., 2013) and learning in many other domains (see Chein and Schneider, 2005 for review). In contrast to increased activity in the working memory network (WMN) being associated with top-down modulation of attention and working memory, the default-mode network (DMN) may decrease its activity when attention is focused on an external task. Insight into the function of the DMN derives from two types of study, one in which participants undertake self-referential tasks such as those involving autobiographical memory, thinking about one’s future, theory of mind and affective decision making (Ochsner et al., 2004; Buckner et al., 2008; Spreng et al., 2008), and other studies involving a passive or resting state, when participants are not engaging with a specific task (McKiernan et al., 2003; Mason et al., 2007; Christoff et al., 2009). When participants are supposed to be engaged with a task, the DMN has been observed to activate when attention is shifted away from the task toward unrelated internal thoughts and feelings, i.e., during the act of mind-wandering (Smallwood and Schooler, 2006; McVay and Kane, 2010). Mind wandering may not always be wholly unhelpful, since it can support autobiographical planning and creative problem solving (Mooneyham and Schooler, 2013). However, it is associated with a failure to encode (Otten and Rugg, 2001; Wagner and Davachi, 2001; Daselaar et al., 2004, 2009) and to difficulties in subsequent comprehension when it occurs during reading (Schooler et al., 2004; Smallwood et al., 2008; Unsworth and McMillan, 2013). It might, therefore, be assumed antithetical to most notions of educational engagement. Andrews-Hanna et al. (2010) have classified some DMN regions as subsystems, with a dorsal medial prefrontal subsystem preferentially engaged for participants’ self-referential judgements about the present and a medial temporal lobe subsystem by episodic judgements about their personal future. In contrast, their review identified the anterior medial prefrontal cortex (aMPFC) and posterior cingulate cortex (PCC) as core components which activated in both such conditions and were selected as the focus of DMN analysis in the present study. The putative opposite effects of engaging with an external task on the WMN and DMN may explain why anticorrelation of these two networks has been frequently reported (Greicius et al., 2003; Fox et al., 2005; Fransson, 2005; Weissman et al., 2006; Buckner et al., 2008; Christoff et al., 2009; Panda et al., 2014), and has resulted in them being dubbed the “task-positive” and “task-negative” networks (Fox et al., 2005).

In light of the above arguments, if gamification increases engagement with a goal-directed educational learning task (without additional self-referential or creative processing), we might expect to observe increased WMN activity and decreased DMN activity with gamification. Such predictions could be tested using a well-theorized learning game environment designed to engage its player. The mechanisms by which games, including learning games, incentivise their players remain to be fully understood, but some insights can be provided by our emerging understanding of the reward system. Midbrain dopamine neurons which project to the ventral striatum (VS) fire in response to cues that predict reward (Schultz, 1998) and during anticipation of reward (Fiorillo et al., 2003), with fMRI research indicating that VS activation increases in proportion to the magnitude of anticipated reward (Knutson et al., 2001). In this way, VS activation provides a potential index for motivational state and an increase in VS response has been reported when adults play off-the-shelf video games (Koepp et al., 1998; Hoeft et al., 2008; Weinstein, 2010; Lorenz et al., 2015). Activation of this dopaminergic pathway has been shown to predict declarative memory formation, and an estimate of VS response has been shown to predict correct answers in an educational learning game (Howard-Jones et al., 2011). The features of a game that contribute to activation of this midbrain dopaminergic response and its potential role in cognition are the subject of nascent research, but the schedule of rewards that games offer their players may play an important role in their power to engage and to influence our learning (Howard-Jones and Demetriou, 2009; Bavelier et al., 2010; Howard-Jones et al., 2011). For example, games often escalate rewards and so challenge expectations in a positive way. A strong relationship between midbrain dopaminergic response and prediction error (Schultz et al., 1997; Schultz and Dickinson, 2000) suggests this scheduling may help sustain greater phasic responses to anticipated rewards than offering the same value of reward throughout. Also, since game players find playing with or against each other more engaging and enjoyable (Gajadhar et al., 2008), the reported role of peer-presence on reward system activity (Chein et al., 2011) may also be a factor. Additionally, it has been suggested that the uncertain nature of reward in all games may increase the brain’s response to the anticipated reward (Howard-Jones and Demetriou, 2009). In primate and human studies (Fiorillo et al., 2003; Preuschoff et al., 2006), the uncertainty of a reward has been shown to increase the release of midbrain dopamine into the VS, helping to explain our preference for uncertain rewards our attraction to games (Shizgal and Arvanitogiannis, 2003). This joins other behavioral reports of improved learning when rewards are uncertain (Ozcelik et al., 2013; Devonshire et al., 2014) to suggest increased reward system activity may underlie the supposed educational advantages of gamification. These three factors (competition, escalation and uncertainty) characterize the Game-based learning context investigated here (Howard-Jones et al., 2014a). We do not, of course, claim that our Game-based condition can be considered representative of all games. Rather, the context we attempted to create in this condition comprised a particular configuration of a small set of features common to many popular games, and for which we have some theoretical rationale for considering may contribute to engagement and learning. We would also emphasize that the evidence base for theorizing the design of learning games is very incomplete and the underlying neural and cognitive processes are poorly understood. Indeed, there have been no previous attempts to physiologically measure reward system response in educational learning games or even, to our knowledge, in any educational learning task.

To explore how “gamification” of a learning context might influence engagement and learning, we used fMRI to measure brain activity when adults were studying in three conditions in a within-participants design. Our design also allowed the educational learning achieved between conditions within a defined time period (a learning window) to be meaningfully compared. In this way, it provided an indication of the comparative efficiency of the three approaches, which has been identified as an important issue for those interested in evaluating the effectiveness of digital game-based learning (All et al., 2015). The three conditions were a *Study-only* condition in which learning material (comprising text and images) was presented during the learning window, followed by an exemplar question and answer, a *Self-quizzing* condition in which each learning window was followed by a question that the student must answer in return for a fixed number of points, and a *Game-based* condition in which the learning window was followed by a question which the participant competed with a peer to answer correctly, with an escalating and uncertain number of points as a reward. The Self-quizzing condition was intended to represent a level of gamification between that of the Study-only and Game-based conditions. This self-quizzing condition required the participant to test themselves, as in a solitary quiz, but omitted the factors of competition and escalating and uncertain rewards. In behavioral terms, we hypothesized a measurable increase in learning, as measured by pre-test/post-test, as the context became more gamified (Study-only < Self-quizzing < Game-based). Alongside this behavioral increase with gamification, we hypothesized greater activation of WMN and deactivation of DMN during learning, and greater increases in ventral striatal activity when responding to questions and when receiving positive feedback.

## Materials and Methods

This study was carried out in accordance with the ethical procedures of the University of Bristol (Graduate School of Education) with written informed consent from all subjects. All subjects gave written informed consent in accordance with the Declaration of Helsinki.

### Participants

Informed consent was obtained from 24 healthy student volunteers (6 males, 18 females) who responded to an advertisement placed in the Education Department of the University of Bristol. The department’s population has a strongly international profile and the study was conducted in both Spanish and English to facilitate recruitment. The first 12 volunteers in each language category who met criteria (i.e., right-handed, no metal, no psychoactive medications) were recruited. The mean age of participants was 34.7 years, *SD* = 7.6 years.

### Stimuli

For the sake of ecological validity, learning corpora were constructed that addressed a diverse range of educational topics (including history, biology, mathematics, grammar, electronics, music, horticulture). Four corpora were developed, each consisting of 10 topics that were exclusive to that corpus. For each topic, learning content was generated that consisted of a screen of text and pictures, along with an associated pair of questions (resulting in 10 screens of content and 20 questions for each corpus). Corpora (content and questions) were designed to provide an educational challenge that extended beyond factual recall. To achieve this, each of five levels of educational learning objective, as defined in educational terms by Bloom’s taxonomy (Bloom, 1956), were represented within each corpus by two pairs of questions. In this way, each corpus focused four questions on each of five of the six educational learning objectives defined by Bloom (*remembering, understanding, applying, evaluating*, and *analyzing*). The exception was *creativity* which was omitted due to difficulties in assessing this type of learning objective in the present experimental paradigm.

The four corpora were permutated across the three scanning conditions of each participant, with the remaining corpus being reserved for the Game-based condition when they competed with their partner as their partner was being scanned. In this way, each participant encountered each learning corpus only once. Additionally, within each subgroup (*N* = 12) of Spanish and English speakers, and within each presentation position (first, second, third), each condition was combined once only with each corpus.

Three different sets of 40 multiple-choice questions (120 questions) on the learning content were also generated. These were designed to test participants knowledge and understanding of the learning content immediately before (pre-test) and after (post-test) being scanned and following a period of 3–4 weeks (retention test). Each set comprised 1 question on each of the 10 screens of content within each of the four corpora. These were novel questions, in the sense that they were similar in form, but did not replicate, the questions that participants experienced during scanning. The three sets were allocated for use as pre-, post-, and retention tests, with balanced permutation of question set across the three types of test within each subgroup of Spanish and English speakers.

### Task and Conditions

**FIGURE 1:**
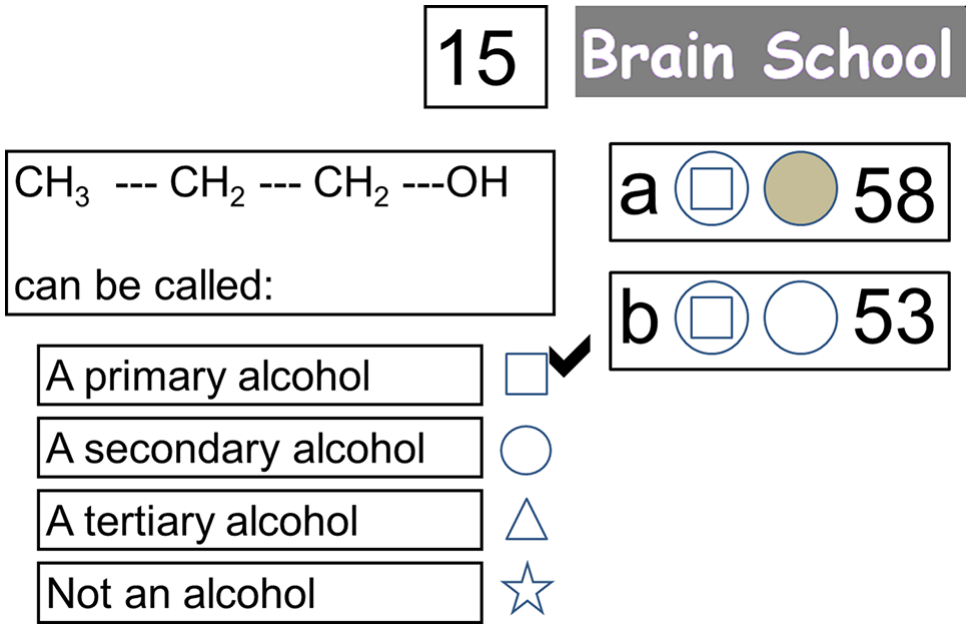
**Screen shot of interface when participants are receiving feedback on their response to the question (with the correct answer now indicated with a tick).** The response (as shown by the symbol in the first circle) of both the participant being scanned (player “a”) and their competitor in the control room (player “b”) was correct. Since the participant being scanned had decided to game their points (as shown by the filled second circle) he/she may now win 30 points or no points depending on a wheel of fortune that is about to appear. Their competitor did not decide to game their points, so they will receive 15 points for their correct answer irrespective of the wheel of fortune.

**FIGURE 2:**
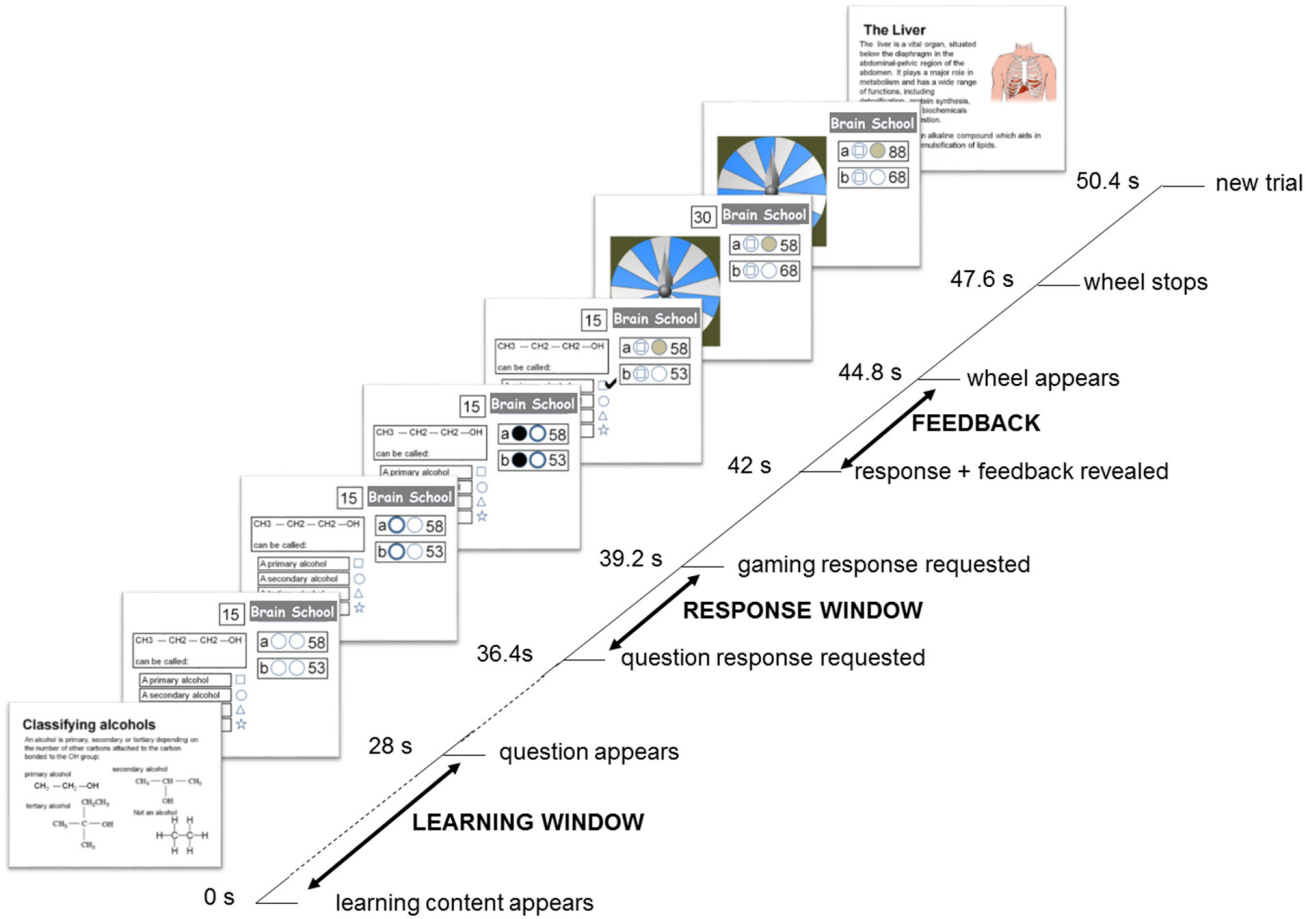
**Trial timeline for the Games-based condition, with main epochs of interest indicated in capitals.** In this condition, participants were asked to study the learning content carefully (which was presented twice in the block to encourage rehearsal, but always with a novel question) before answering a question in return for points that could be doubled or lost on a wheel of fortune. At the same time, a competitor in the control room was trying to accumulate points in the same way. Responses of participant and competitor were hidden from each other until the correct answer was revealed, and both were required to decide whether they would be gaming on the wheel of fortune, should their answer be correct, before this outcome was known. The points available for a correct answer escalated over the block from 1 to 19. The duration of each trial was 50.4 s resulting in each the three conditions of 20 trials lasting 16 min and 48 s and, allowing for a momentary pause between conditions, a total functional scanning time of approximately 51 min.

Participants experienced three experimental conditions that represented three learning contexts (Study-only, Self-quizzing, and Game-based). Following study of each topic, the Study-only condition required participants to observe an exemplar question and answer, the Self-quizzing condition required them to select an answer for the question, and the Game-based condition required them to compete with a friend to select an answer, and to game their potential points on a wheel of fortune. These conditions were implemented using an interface resembling that used by Zondle Team Play (Zondle, 2013), an online app that is used by teachers to facilitate whole-class learning games (see **Figure 1**). At the beginning of each condition, scores were set at zero. At the beginning of each trial (featuring a new question) participants were first presented with the learning content required to answer the question for 28 s (see **Figure 2**). After this time, the interface would appear that displayed the multiple-choice question. The interface also showed the number of points available for the trial (or question), and the current score for the participant and (when present) their competitor. Two circles were shown in front of the participant’s current score. The first circle related to the participant’s response to the question, the second related to their decision to “game” their points in the Game-based condition. After the question had been displayed for 8.4 s, the first circle illuminated for up to 2.8 s, signaling that the participant must respond to the question. During this period, the participant (and, if present, their competitor) was required to press one of four buttons to indicate their preferred response to the question, causing the first circle to change the color of its interior to gray, indicating a response had been made. The participant held two of the buttons (for choosing the first and second response options) in a box in their left hand, and two buttons (for choosing the third and fourth response options) in a box in their right hand. The first circle’s illumination disappeared at the end of this 2.8 s period, indicating that it was now too late to respond.

In the Study-only condition, participants were presented with a question accompanied by only one answer option (the correct response). To balance conditions and ensure wakefulness, participants were still required to select this answer, after which their score would increase by 10 points.

In the Game-based condition, each of the four options featured a plausible response to the question, and participants were asked to choose the correct response during the 2.8 s question response window. In this condition, at the end of this period, the second circle would illuminate. This indicated that the participant and their competitor had 2.8 s to press the first button held in their left hand again, should they wish to see their winnings for a correct answer “gamed” on the wheel of fortune, and not press it if they did not wish to game their points. At the end of this gaming decision window, the response of both participant and their competitor to the question were revealed (inside the first circle) and the correct response was marked with a tick. At the same time, a participant and/or competitor who had not decided to game their points but had answered correctly would see their score increased by the points available for the question. After 2.8 s of displaying this information, a spinning wheel of fortune appeared. At the end of a further 2.8 s, the wheel of fortune stopped on either blue or white (with 50% probability). For both the participant and competitor, a correct response and prior commitment to game their points would result either in gaining double the points available for the round if the wheel landed on blue, or in losing their points for the round if it landed on white. The outcome of the spinning wheel of fortune remained on the screen for 2.8 s, before the next trial began. In this condition, points began with one point for the first two questions and increased by two points every two questions such that, over a 20 trial block, 19 points were offered for the last two questions (averaging 10 points per question – as in the other conditions). The order in which screens of learning content appeared was automatically randomized for each participant, with each screen appearing twice with a block, but always with a new question. Participants were made aware that, all else being equal, the average value of gaming was equivalent to the value of not gaming.

The Self-quizzing condition was identical to the Game-based condition except that no competitor was present and 10 points were provided for selecting the correct response. The average value of points for correct responses across the three conditions was, therefore, equivalent.

To ensure equal durations of trials in all conditions, a 2.8 s rest was provided in the Study-only and Self-quizzing conditions instead of a gaming decision window. A spinning wheel of fortune still appeared in the Study-only and Self-quizzing conditions for 2.8 s, but it was made clear to participants that this had no implication for their scores.

### Procedure

An advertisement requested volunteers to apply for “Brain School,” in which successful candidates would have the opportunity to learn some interesting general knowledge while having their brain scanned. Candidates were called to attend a preliminary meeting with researchers in pairs. They completed an initial survey of medical history to ensure they met scanning criteria, and the task and three conditions were then explained to them. Both candidates then experienced a shortened (15 min) version of the procedure together with fMRI simulation, each competing with the other candidate in the pair during the Game-based condition. Each applicant experienced two trials in each condition, using content and questions not employed in the main scanning experiment. This simulation helped ensure volunteers were aware of the procedures they were consenting to, and also helped acclimatize them to the scanning environment. Pairs who passed safety criteria and were able to provide informed consent for scanning were then offered a slot on one of four scanning days.

On arrival at the scanner, each member of a participating pair completed a 40 question pre-test (featuring 10 novel questions for each corpus) which assessed their prior knowledge of the material they would be learning. Inside the scanner, one member of each pair was functionally scanned while experiencing all three conditions in quick succession, with each condition comprising a block of 20 trials. In Study-only trials, participants simply had to study each slide of learning content, and the exemplar question and answer that followed it, acknowledging the latter with a press of a button. In the Self-quizzing condition participants were required to choose an answer for the question from four options, receiving 10 points for a correct response. In the game-based condition, the other member of the pair (as competitor) was also responding to this question from the control room, with both participant and competitor winning points for correct answers that escalated (from 1 to 19) over the block and that could be doubled or lost on a wheel of fortune. Following the three conditions, a structural scan was completed before the participant left the scanner. The pair of participants then swapped their roles, with the other member of the pair experiencing three conditions while being scanned and their partner acting as competitor in the Game-based condition. When scanning was completed for both members of the pair, the participants individually provided a brief self-reported evaluation of conditions in terms of engagement and stress, and were then asked to complete a 40 question post-test. Self-reports of engaged and stressed participants felt were indicated on a 5-point Likert scale, where 1 was labeled “not at all” and five was labeled “extremely”. After a period of 3–4 weeks, participants were recalled for another 40 question retention test.

### fMRI Image Acquisition

All of the images were collected on a Siemens 3T Magnetom Skyra MRI scanner at the Clinical Research Imaging Centre (CRIC) at the University of Bristol. The functional images were collected using an echoplanar T2^∗^ gradient-echo EPI sequence to measure BOLD contrast over the entire brain (36 contiguous 3 mm-thick axial slices; *TR* = 2800 ms; *TE* = 30 ms; flip angle = 90°; in-plane voxel size = 3 mm × 3 mm). A T1-weighted anatomical dataset was obtained from each participant (*TR* = 1.800 ms, *TE* = 2.25 ms, *FA* = 9°, *Fov* = 240 mm × 240 mm, *ST* = 0.90 mm, spatial resolution 0.90 mm × 0.90 mm × 0.90 mm).

Visual stimuli were presented with a personal computer running in-house software implemented in Visual Basic (Microsoft), back projected onto a translucent screen and viewed through a mirror attached to the head coil. The presentation timing was controlled and triggered by the acquisition of the fMRI images.

### fMRI Data Analysis

The functional MRI data were pre-processed and analyzed oﬄine using SPM8 (Wellcome Department of Cognitive Neurology, Institute of Neurology, London). For each subject, the functional images were first spatially corrected for head movements using a least-squares approach and six-parameter rigid-body spatial transformations (Friston et al., 1995). The realigned functional images were then corrected for differences in the timing between slices, using the middle slice acquired in time as the reference. The high-resolution anatomical image and the functional images were co-registered and then stereotactically normalized (using trilinear interpolation) to the Montreal Neurological Institute (MNI) brain template used in SPM8 (Mazziotta et al., 1995). The functional images were re-sliced with a voxel size of 3 mm × 3 mm × 3 mm and spatially smoothed with a three-dimensional Gaussian filter with a 12-mm full-width at half maximum to accommodate anatomical variations between subjects (Friston et al., 1995).

The images were subsequently analyzed using a random-effects approach. At the first stage, the time series of the functional MR images obtained from each participant were analyzed separately. The effects of the experimental paradigm were estimated on a voxel-by-voxel basis, according to the general linear model extended to allow the analysis of fMRI data as a time series (Worsley and Friston, 1995). The three key epochs of interest were the learning window (28 s, or 10 scans duration), the question response window (2.8 s, or 1 scan duration) and the period when question feedback was provided (2.8 s, or 1 scan duration). However, to allow measurement of change in BOLD during the learning window, this was further divided into five sub-periods (each consisting of 5.6 s of data, or 2 scans), creating a total of seven epochs of interest per trial. Time points at each of these seven epochs (learning window as five sub-periods, response window, question feedback) were further divided into correct and incorrect trials, creating 14 regressors for each of the three conditions. In these first level analyses, individual BOLD data was modeled with boxcar stimulus functions convolved with a canonical hemodynamic response function to form these regressors. These single-participant models were used to compute three contrast images (Self-quizzing vs. Study-Only, Game-based vs. Self-quizzing and Game-based vs. Study-only) for each of the seven epochs (learning window as five sub-periods, response window, question feedback), with the contrast for the response window arranged to compare trials with positive feedback to those with negative feedback.

For inference at group level, these contrasts were subjected to a second level analysis in which random effects group statistics were generated. Regions of interest (ROIs) were *a priori* defined as spheres of 7 mm radius at locations identified in previous studies of the WMN and DMN. For the WMN, spherical ROI’s of 7 mm radius were selected at locations identified by Koshino et al. (2014) during preparation and execution periods of a verbal working memory task. These were in the left (-42, 34, 19) and right Dorsolateral Prefrontal Cortex (DLPFC) (40, 34, 21) and a posterior inferior (BA40) region of the parietal lobe in left (-46, -48, 40) and right Inferior Parietal Lobule (IPL) (43, -43, 42). Of the many regions that have been associated with the DMN, the aMPFC, and PCC are of particular significance, since these have been considered to represent the major hubs of the DMN (Andrews-Hanna et al., 2010). Therefore, for the DMN network, spherical ROI’s of 7 mm radius were selected at locations of aMPFC [(-7, 50, 14), (5, 50, 14)] and PCC [(-7, -51, 26), (4, -51, 25)] as also identified by Koshino et al. (2014). To test hypotheses regarding learning in the three conditions, contrasts were first calculated to detect changes across the whole learning window in WMN and DMN activity in the Game-based condition compared to the Self-quizzing and Study-only conditions, and in the Self-quizzing condition compared to the Study-only condition. In contrasts where hypotheses were upheld, a further analysis explored changes in parameter estimates in the five sub-periods comprising the learning window. The marsbar toolbox for SPM (marsbar.sourceforge.net/) was used to extract parameter estimates within functional ROIs. To test hypotheses regarding ventral striatal activity during answering and feedback, spherical ROIs (6 mm radius) were defined at the location of the Nucleus Accumbens (NAcc) at (±10, 6, -4), as reported on the basis of anatomical and functional studies (Neto et al., 2008), and converted to MNI coordinates using the Brett transform (Brett et al., 2001). ROI analyses for all contrasts were thresholded at *p* < 0.05, family wise error (FWE) using small volume correction. For completeness, all contrasts were also explored using whole brain analyses and these are reported at *P*_fwe_(whole-brain) = 0.05 with an extent threshold of 10 voxels (Forman et al., 1995). Images of activity are displayed at *p* < 0.001 uncorrected with an extent threshold of 10 voxels.

## Results

### Behavioral Results

Behavioral results were analyzed using IBM SPSS Statistics, version 22. The average number of questions correctly answered by participants during the self-quizzing and game-based conditions were similar (see **Table 1**) and a paired *t*-test did not reveal significant differences when a comparison was made of participants’ responses in these two conditions [*t*(24) = 0.429, *p* = 0.672]. Independent samples *t*-tests did not reveal any significant difference in the number of correct responses made by participants and competitors in the game-based condition [*t*(46) = 1.60, *p* = 0.496], or in the percentage of decisions to game points following a correct answer in this condition [*t*(46) = 0.933, *p* = 0.929].

**Table 1:** Descriptive statistics of percentage correct scores achieved by individuals during learning in the Self-quizzing and Game-based conditions, and for decisions to game following correct responses in the Game-based condition (*N* = 24 in all cases).

		*M*	*SD*
Self-quizzing	% Questions answered correctly by participants	51.4	13.9
Game-based	% Questions answered correctly by participants	49.8	14.7
	% Questions answered correctly by competitors	46.0	14.1
	% Occasions participants chose to game points	65.9	28.2
	% Occasions competitors chose to game points	73.7	29.6

Means and standard deviations of the pre-test, post-test and retention scores for learning content experienced in each of the three conditions are provided in **Table 2** and suggest participants generally found learning in each condition suitably challenging. Measures of immediate learning and retained learning were calculated as, respectively, the increase in post-test and retention test scores relative to the pre-test score. Descriptive statistics for these measures are provided in **Table 3**. A 3 × 2 factorial repeated measures ANOVA was applied to investigate effects of condition (three levels: Study-only, Self-quizzing, and Game-based) and the time of test (two levels: immediate learning and retained learning after 3–4 weeks). Mauchly’s test showed that the condition of sphericity had been met, χ^2^(2) = 1.60, *p* = 0.450. Main effects were found for condition (*F*_2,46_ = 3.69, *p* = 0.033) and time of test (*F*_1,23_ = 61.57, *p* < 0.001) with no significant interaction between condition and time (*F*_2,46_ = 0.313, *p* = 0.733). Planned contrasts were carried out, collapsed across time, where learning in the Game-based condition was found to be significantly higher than that for the Study-only and Self-quizzing conditions together (*F*_1,23_ = 5.86, *p* = 0.024), while there was no difference between the Study-only and Self-quizzing conditions (*F*_1,23_ = 2.02, *p* = 0.169).

**Table 2:** Descriptive statistics for scores (out of 10) achieved in the pre-test, post-test, and retention tests (*N* = 24 for each type of test) for topics covered in each of the three conditions (study-only, self-quizzing, and game-based).

	Pre-test		Post-test		Retention test	
	*M*	*SD*	*M*	*SD*	*M*	*SD*
Study-only	3.92	1.53	5.50	1.77	3.63	1.93
Self-quizzing	3.42	1.64	5.58	2.00	4.17	1.53
Game-based	3.21	1.69	6.25	1.82	4.38	1.55

**Table 3:** Descriptive statistics of measures of immediate and retained learning calculated as the increase in post-test and retention test scores relative to the pre-test score.

	Immediate learning		Retained learning	
	*M*	*SD*	*M*	*SD*
Study-only	1.58	1.86	-0.29	2.53
Self-quizzing	2.17	2.50	0.75	2.13
Game-based	3.04	2.48	1.17	1.97

*The post-test was completed immediately after leaving the scanner, and the retention test was completed 3–4 weeks after the scan.*

Looking at the two time points separately using one-way ANOVAs, immediate learning was not significantly different across conditions (*F*_2,46_ = 2.56, *p* = 0.088), and neither was retained learning following a delay (*F*_2,46_ = 2.95, *p* = 0.062), although both approached significance.

A 3 × 2 × 2 (condition × time × language) ANOVA revealed no additional main effect of first language (*F*_1,22_ = 0.178, *p* = 0.678) and no interaction between first language and either experimental condition (*F*_2,44_ = 0.682, *p* = 0.478) or time (*F*_1,22_ = 1.046, *p* = 0.317). Assumptions of sphericity were verified using Mauchly’s test as before, χ^2^(2) = 1.60, *p* = 0.450.

Across participants, the means of self-reported measures of engagement and stress were all in the direction Game-based > Self-quizzing > Study-only (see **Table 4**). Within-participants analyses showed main effects for self-reported engagement [*F*(2,46) = 77.1, *p* < 0.001] and stress [*F*(2,46) = 39.72, *p* < 0.001] across conditions.

**Table 4:** Means (with standard deviations in parentheses) of self-reported measures of engagement and stress (on a scale of 1–5) for the three conditions (Study-only, Self-quizzing, Game-based) reported immediately following scanning.

	Engagement		Stress	
	*M*	*SD*	*M*	*SD*
Study-only	3.83	2.31	2.54	1.98
Self-quizzing	7.33	1.43	5.54	1.86
Game-based	8.7	0.93	6.88	2.40

In each condition, positive associations were sought between self-rated engagement and the learning achieved, and negative associations between self-rated stress and the learning achieved (see **Table 5**). Only correlation between self-rated engagement and learning in the Game-based condition reached statistical significance (Pearson’s *r* = 0.583, *p* = 0.007).

**Table 5:** Correlation statistics (Pearson’s *r)* for associations between self-reported engagement and stress in each condition with measures of subsequent immediate and retained learning (*N* = 24 in all cases, ^∗∗^statistically significant at *p* < 0.01).

		Correlation with subsequent measures of learning			
		Immediate		Retained	
		Pearson’s *r*	Significance (2-tailed)	Pearson’s *r*	Significance (2-tailed)
Study-only	Engagement	-0.227	0.285	0.111	0.606
	Stress	-0.029	0.895	0.183	0.404
Self-quizzing	Engagement	0.214	0.315	-0.264	0.213
	Stress	0.052	0.812	-0.082	0.710
Games-based	Engagement	0.533	0.007**	0.171	0.424
	Stress	-0.149	0.497	0.036	0.872

### Imaging Results

#### Learning Window

No significant increases in activity in WMN ROIs during the learning window were identified when comparing the Self-quizzing condition with the Study-only condition, or the Game-based condition with either the Self-quizzing or the Study-only condition. A whole brain analysis revealed no unhypothesised activations at *P*_fwe_(whole-brain) < 0.05 for these contrasts.

**FIGURE 3:**
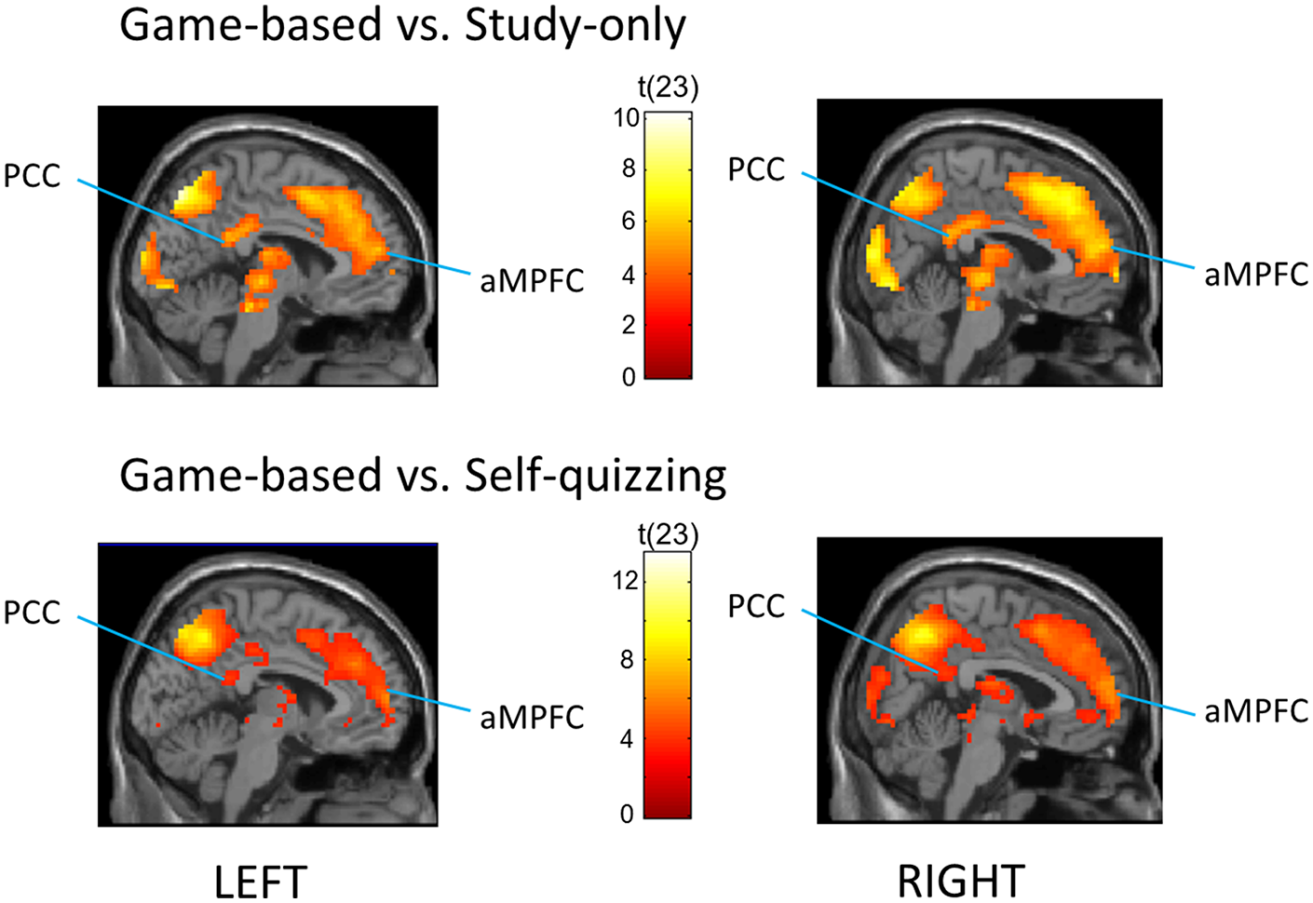
**Regions that showed greater deactivation in the Game-based condition compared with (TOP) the Study-only and (BOTTOM) the Self-quizzing condition, including bilateral Posterior Cingulate Cortex (PCC) and anterior Medial Prefrontal Cortex (aMPFC).** The image is thresholded at *P* < 0.001 uncorrected and an extent of 10 voxels, with peak coordinates at Pfwe(whole-brain) < 0.05 provided in **Tables 2** and **3**.

Default mode network ROIs significantly deactivated in the Game-based condition compared with both the Study-only and the Self-quizzing conditions (see **Figure 3**). When comparing activity during the learning window in the Game-based condition with the Study-only condition, left and right aMPFC were significantly deactivated [*P*_fwe_(SVC) < 0.001 for left and right] and also left and right PCC [*P*_fwe_(SVC) = 0.004, *P*_fwe_(SVC) = 0.002, respectively]. When comparing activity during the learning window in the Game-based condition with the Self-quizzing condition, left and right aMPFC were significantly deactivated [T_(23)_ = 4.74, *P*_fwe_(SVC) = 0.001 and T_(23)_ = 6.23, *P*_fwe_(SVC) < 0.001, respectively] and also left and right PCC [T_(23)_ = 3.62, *P*_fwe_(SVC) = 0.013 and T_(23)_ = 4.68, *P*_fwe_(SVC) = 0.002, respectively]. No differences in deactivation in any these DMN ROIs could be identified in the Self-quizzing condition relative to the Study-only condition [at *P*_fwe_(SVC) < 0.05].

A whole-brain analysis at *P*_fwe_(whole-brain) < 0.05 was carried out (see **Table 6**) for the Game-based condition compared with the Study-only condition. This identified a large cluster of occipital-parietal activation with a center in the left cuneus/precuneus [(*x* = 12, *y* = -94, *z* = 16): *T*_(23)_ = 10.06; *P*_fwe_ < 0.001], and extending from bilateral extrastriate cortex (BA 18/19) to include left and right IPL. A large cluster of right medial gyrus activity was identified (BA 6/8; *x* = 6, *y* = 32, *z* = 46): peak [*T*_(23)_ = 7.70, *P*_fwe_ = 0.001], together with activations in bilateral inferior frontal, middle frontal and superior temporal gyri, and right thalamus.

**Table 6:** Regions deactivating for the learning window in the Game-based compared with Study-only condition [*P*_fwe_(whole brain) < 0.05, extent threshold 10 voxels].

Region		L/R	BA	Peak coordinates (MNI)	Peak *Z* score	Size in voxels
Cuneus	1	R	18	12 -94 16	6.21	1955
Precuneus	2	L	7	-6 -73 52	6.17	
Cuneus	3	R	19	18 -94 22	6.05	
Inferior frontal gyrus	4	R	9	48 11 34	6.09	386
Middle frontal gyrus	5	R	6	36 -1 52	5.49	
Middle frontal gyrus	6	R	9	45 26 31	5.17	
Inferior frontal gyrus	7	R	47	51 20 -2	5.37	88
Inferior frontal gyrus	8	R	47	33 20 -11	4.99	
Medial frontal gyrus	9	R	8	6 32 46	5.36	285
Medial frontal gyrus	10	R	6	6 14 52	5.36	
Medial frontal gyrus	11	R	6	6 38 40	5.09	
Inferior frontal gyrus	12	L	47	-33 17 -11	5.32	67
Temporopolar cortex	13	L	38	-48 17 -11	5.23	
Lingual gyrus	14	L	19	-27 -73 -8	4.97	19
Thalamus	15	R		9 -16 10	4.90	19
Supramarginal gyrus	16	R	40	57 -49 25	4.89	21
Superior temporal gyrus	17	R	13	57 -49 16	4.59	
Middle frontal gyrus	18	L	6	-27 -7 52	4.87	18
Red nucleus	19	L		0 -22 -5	4.72	10

An analysis at *P*_fwe_(whole-brain) < 0.05 was also carried out to detect deactivations across the whole brain for the Game-based condition compared with the Self-quizzing condition (see **Table 7**). In addition to a broad prefrontal right-hemisphere cluster with centers on middle and superior frontal gyri, this analysis identified two large clusters of activity with bilateral centers in occipital cortex (BA19), both extending to right and left inferior parietal cortex.

**Table 7:** Regions deactivating for the learning window in the Game-based compared with Self-quizzing condition [*P*_fwe_(whole brain) < 0.05, extent threshold 10 voxels].

Region	L/R	BA	Peak coordinates (MNI)	Peak *Z* score	Size in voxels
Inferior temporal gyrus	R	19	45 -73 1	7.03	2790
Superior parietal lobule	R	7	24 -73 46	6.34	
Precuneus	R	7	18 -70 53	6.30	
Middle occipital gyrus	L	19	-45 -76 4	6.78	330
Middle occipital gyrus	L	19	-27 -88 16	5.03	
Superior frontal gyrus	R	6	21 14 49	5.62	585
Middle frontal gyrus	R	9	48 14 34	5.51	
Middle frontal gyrus	R	8	39 23 40	5.50	
Precentral gyrus	L	9	-33 23 37	4.76	11

Whole-brain analyses failed [at *P*_fwe_(whole-brain) < 0.05] to detect increased activations during the learning window in the Game-based or Self-quizzing conditions relative to the Study-only condition, or with respect to the Game-based condition relative to the Self-quizzing condition.

**FIGURE 4:**
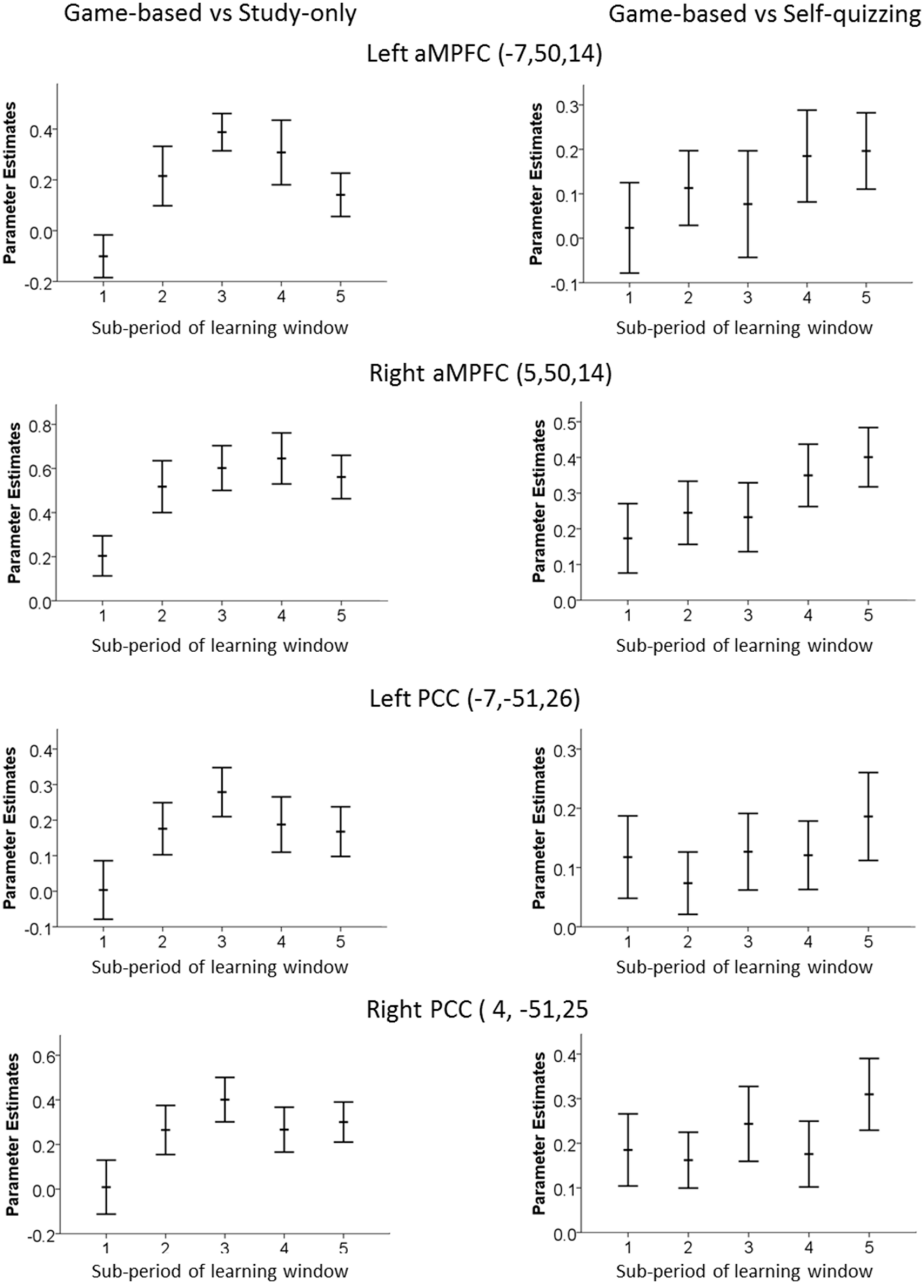
**Time-variation of Default Mode Network ROI in the left and right aMPFC and PCC.** Graphs were created by analyzing each 5.6 s sub-period of the 28 s learning window in the game-based condition and show the extent of decrease relative to **(left)** the Study-only condition and **(right)** the Self-quizzing condition. An increase in the parameters estimates plotted here represents greater deactivation.

Trials in the present study were phase-locked to the scanner repetition time and not jittered. This allowed a pace of delivery and more trials, but prevented estimation of the BOLD timecourse on a time scale of fractions of seconds. Instead, a grosser estimate of change in BOLD response was obtained from contrasts for each of the five sub-periods of the learning window, to show how the level of deactivation of DMN ROIs changed between these 5.6 s periods. These contrasts were calculated for the Game-based condition (which produced greatest DMN deactivation) compared with the Study-only condition, and compared also with the Self-quizzing condition. These are shown in **Figure 4**. The apparent dip in DMN activity in the later part of the learning window in the Game-based vs. Study-only was explored by making a paired comparison of the combined activities over the four regions for each participant at sub-period 3 and 5, revealing a significant diminishment [*T*_(23)_ = 2.09, *p* = 0.047].

#### Responding to Questions

Activity in the VS during the response window in the Self-quizzing and Game-based conditions was calculated by comparing ROI activity in the conditions with the Study-only condition, where a question and answer was presented with no response required.

**FIGURE 5:**
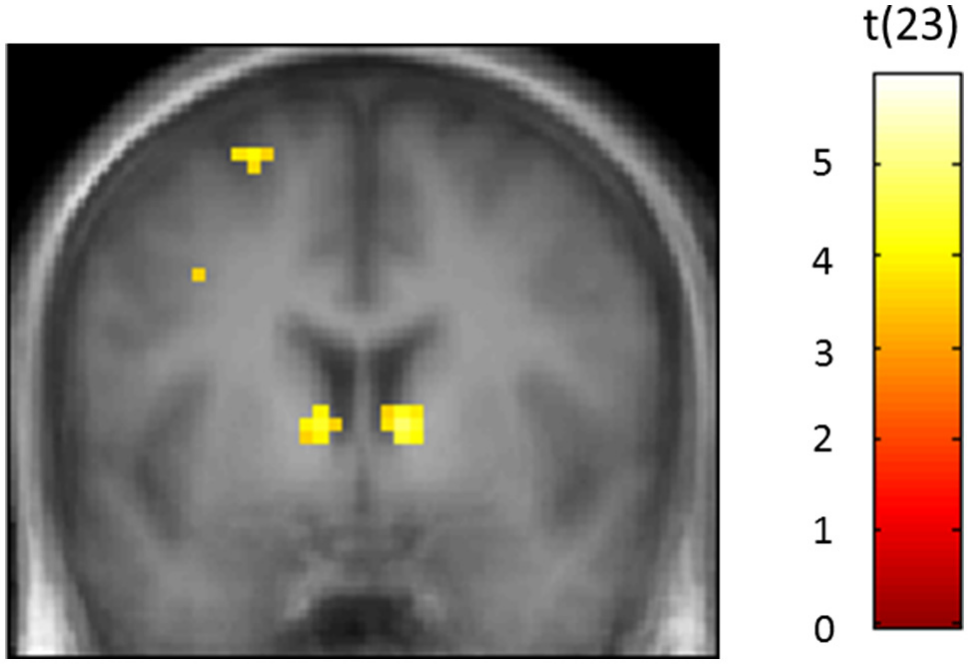
**Bilateral activity in the ventral striatum (VS) when participants were responding to the question in the Games-based condition relative to the Study-only condition.** The Game-based condition required participants to choose the correct answer amongst four options. The Study-only condition presented only one option with the question, which was the correct answer. The image is thresholded at *P* < 0.001 uncorrected and an extent of 10 voxels.

Statistically significant activation associated with responding in the Self-quizzing condition, compared with the Study-only condition, was found in the left and right ventral striatal ROIs [*T*_(23)_ = 2.71, *P*_fwe_(SVC) = 0.046 and *T*_(23)_ = 3.57, *P*_fwe_(SVC) = 0.009, respectively]. Activation associated with responding in the Game-based condition, relative to the Study-only condition, was also identified in both the left and right ventral striatal ROIs [*T*_(23)_ = 4.28, *P*_fwe_(SVC) = 0.002 and *T_(_*_23)_ = 4.72, *P*_fwe_(SVC) < 0.001, respectively), see **Figure 5**]. A comparison of Self-quizzing and Game-based conditions did not reveal statistically significant differences in left or right ventral striatal activation [*T*_(23)_ = 1.62, *P*_fwe_(SVC) = 0.201 and *T*_(23)_ = 0.40, *P*_fwe_(SVC) = 0.507, respectively].

#### Feedback

For positive, compared with negative feedback, there was increased activity in left and right ventral striatal ROIs in the Game-based condition during feedback [*T*_(23)_ = 3.98, *P*_fwe_(SVC) = 0.004 and *T*_(23)_ = 2.81, *P*_fwe_(SVC) = 0.037, respectively]. Left ventral striatal activity could be detected in the Self-quizzing condition [*T*_(23)_ = 3.05, *P*_fwe_(SVC) = 0.035] for positive compared to negative feedback, but activity in the right ventral striatal ROI did not reach statistical significance [*T*_(23)_ = 2.64, *P*_fwe_(SVC) = 0.054]. A factorial analysis (condition × feedback) did not reveal statistically different levels activity in relation to positive over negative feedback, for the Game-based compared with the Self-quizzing condition in either left or right VS [*T*_(23)_ = 0.61, *P*_fwe_(SVC) = 0.498 and *T*_(23)_ = 1.67, *P*_fwe_(SVC) = 0.223, respectively].

#### Individual Differences

For the Game-based condition, positive associations across participants were sought between deactivation of DMN ROI’s (relative to the quizzing condition) and the immediate learning achieved (relative to the quizzing condition), as calculated by pre-test/post-test difference. Deactivation of the left and right PCC was found to be correlated with learning (Spearman’s *rho* = 0.455, *p* = 0.012 and Spearman’s *rho* = 0.372, *p* = 0.036, respectively), with deactivations in medial prefrontal cortex failing to reach significance. However, no such correlations in the Game-based condition were found when neural activity and learner were compared to the Study-only condition.

## Discussion

This experiment focused on the changes in neural activity when participants studied in three environments that were, by becoming progressively more game-like, intended to increase goal-orientation and engagement with a learning task. The to-be-learned material included a range of different types of knowledge and concepts but, on all trials, success required participants to attend carefully to it by reading and understanding. Self-reported engagement improved with gamification, and the Game-based condition produced higher learning scores than the other (less game-like) conditions. All four ROIs corresponding to the nodes of the DMN in bilateral PCC [7 mm radius spheres at (-7, -51, 26) and (4, -51, 25)] and bilateral aMPFC [7 mm radius spheres at (-7, 50, 14) and (5, 50, 14)] deactivated in the Game-based condition relative to both the Study-only and Self-quizzing conditions. Previous reported activation of DMN with off-task behavior (McKiernan et al., 2003; Mason et al., 2007; Christoff et al., 2009) suggests that relative activation of the DMN in less gamified conditions may be associated with the poorer learning achieved in these conditions. This is further supported by our observation that activity in the posterior hubs of the DMN were negatively correlated with learning performance across individuals.

Given behavioral reports of increasing unrelated thoughts and decreasing attentional performance over time (Smallwood et al., 2006; McVay and Kane, 2009), a general increase in activation of the DMN over sub-periods of the learning window was predicted for the less gamified conditions, as might be associated with increasing levels of mind wandering. This profile of increasing activation of the DMN during the learning window was observed for the Self-quizzing condition relative to the Game-based condition. However, the generally larger differences in DMN activation for the Study-only condition relative to the Game-based condition reached a maximum and partly then declined. This “inverted U” shape may reflect participants’ awareness that they were running out of time in the Study-only condition and about to be tested with a question. If so, it would suggest some part of the off-task behavior in the Study-only condition was amenable to conscious control. This possibility was also suggested by the informal reports from our participants, in which several described their conscious awareness of difficulties in staying on-task. When reflecting on the Study-only condition, they spoke of a “struggle,” that it was “difficult to engage,” and “difficult to pay attention.” While theories based on neuronal energy metabolism may suggest a biologically determined component of mind-wandering (Killeen, 2013), reports of meta-awareness of mind-wandering (Franklin et al., 2014) support such amenability to effortful influence. Caution is required, however, when using a supposed neural correlate (DMN activity) as a proxy for behavior (mind-wandering). This is particularly true if there may be differences in meta-awareness of mind-wandering in the two conditions of Game-based and Self-quizzing, since the absence of meta-awareness is associated with stronger DMN activation during mind-wandering episodes (Christoff et al., 2009). Future studies of the effects of different contexts on the trajectory of mind-wandering behavior during study would benefit from including behavioral probes that more directly examined the contents of participants’ minds through experience sampling.

Whole-brain analysis revealed a range of activities that, although not considered core, have also been associated the DMN. These included dorsal medial prefrontal cortex, implicated in one of two DMN subsystems identified by Andrews-Hanna, also left middle frontal gyrus (Laird et al., 2009) and, more controversially (Fox et al., 2015), temporopolar cortex (BA 38) which appears strongly implicated in emotional processing and also ‘theory of mind’ (or mentalizing; Olson et al., 2007). The thalamus has also been included by some as part of the DMN (Beckmann and Smith, 2004; Fox et al., 2005; Fransson, 2005; Mantini and Vanduffel, 2013) and may be involved with switching between mind-wandering and mindfulness (Wang et al., 2014), such as might occur frequently if participants were struggling to attend. Although spontaneous thought during mind wandering has been strongly linked to the DMN, studies have consistently shown it to recruit other, non-DMN regions (Fox et al., 2015). Studies of spontaneous thought during rest have identified right middle frontal gyrus in a study of spontaneous thought during rest (D’Argembeau et al., 2005) also extra-striate cortex and lingual gyrus (BA 18/19; Christoff et al., 2004, 2009) identified as deactivated by the game-based condition in our whole brain analysis (Fox et al., 2015). This condition also deactivated bilateral inferior frontal gyri which is, perhaps, surprising since this region is associated with the type of conceptual processing that might be required to achieve the observed improvement in learning (Binder et al., 2009). However, it can also be activated for reasoning about self-defining memories (D’Argembeau et al., 2014), such as might occur during a drift away from the intended educational significance of the learning content toward unhelpful personal associations (see below).

The lack of increased activation of WMN with gamification was contrary to the proposed anticorrelation of DMN and WMN networks observed in other studies (Shulman et al., 1997; Mazoyer et al., 2001; Wicker et al., 2003). The prediction of increased WMN activity made at the outset of the present study was based on the assumption that greater demands would be made on working memory, as participants engaged more with the educational task. Although the behavioral data (both in terms of self-reported engagement and subsequent learning scores) suggests such additional processing may have taken place, there was no observable increase in WMN activity with gamification. This is despite a notable decrease in DMN activity with gamification, suggestive of decreased mind wandering. In a study of mind wandering that combined fMRI and experience sampling, it has been demonstrated that co-activation of the executive function network (ACC and DLPFC) with the DMN can occur during episodes of mind-wandering (Christoff et al., 2009). The authors of this study point out that executive region involvement in early fMRI reports of mind-wandering may have been obscured, due to the associated decrease in cognitive demand from activation to baseline when comparing activity during highly practiced tasks and/or conditions of ‘rest’ with novel, cognitively demanding tasks. Conflicting reports on the relation between DMN and WMN may derive, at least in part, from differences in how WMN structures are defined since the regions where activity increases with greater working memory load may depend on the type of information involved (e.g., whether arbitrary numbers or social information). However, the DLPFC is perhaps the region most prominently activated when information processing demands are generally high, and is implicated in studies involving number (Crottaz-Herbette et al., 2004; Zhang et al., 2013), verbal and figural (Loose et al., 2006) information, written language (Manenti et al., 2008), and social information (Meyer et al., 2012). Coactivation of DMN and WMN are reported in tasks that might include a significant sense of self, such as planning the future (Spreng and Grady, 2010), the simulation of hypothetical scenarios (Gerlach et al., 2011), the evaluation of creative works (Ellamil et al., 2012), social working memory (Meyer et al., 2012), constructing scenes (Summerfield and Egner, 2009) and preparation for a verbal working memory task (Koshino et al., 2014). It is possible to conceptualize all these processes occurring during different “types” of mind-wandering, resulting in the possibility that a participant can be “off task” and absorbed in self-orientated thoughts but still be experiencing working memory load comparable to more engaged learning. In other words, in the context of the current study, there may have been a drift in interpretation and processing of the learning content away from its intended educational significance toward its relation to more personal concerns, rather than a diminution in processing *per se*.

Gamification of the learning environment was intended to improve goal-orientated motivation by stimulating the reward system. Alongside greater self-reported engagement as the context became more gamified and deactivation of putative hubs of the DMN, we observed that answering questions, and receiving positive feedback, in the two more gamified conditions activated the VS. Greater learning was achieved in the most gamified (Game-based) condition, and individual learning differences in this condition were correlated with deactivation of the posterior DMN hubs. Given the associated role of the DMN with internal thoughts and feelings unrelated to the task at hand, it seems likely that incentivisation may have increased goal-orientation, and so possibly reduced occurrences of mind wandering and improved learning. In the Game-based condition and Self-quizzing conditions, compared with the Study-only condition, bilateral activation of the VS was observed when participants were responding during a test of their knowledge. This may reflect dopaminergic activity in response to anticipated outcome, even though, in this epoch-related study, this activity is being captured with a temporal resolution that is very limited (In this study, we attempted to provide participants with a well-paced learning and gaming experience similar to that which might be provided for educational purposes. This prevented the inclusion of jittering which would enable reconstruction of temporal changes in event-related BOLD response beyond the resolution of the repetition time). If rehearsal of learning occurred simultaneously with such a dopaminergic response, this might have contributed to the greater learning achieved in the more gamified conditions. Activation of this region in response to cues indicating monetary incentives for remembering has been found to have roughly linear relationship with the likelihood of subsequent recall (Adcock, 2006). Unlike the work of Adcock (2006), however, our study did not lend itself to examining direct links between variations of this activation with learning performance, and there was no statistically significant increase in this activity with gamification (i.e., between the Self-quizzing and Game-based conditions). Nevertheless, this observation of reward system activity might still be relevant to theories seeking to explain the educational benefits of games in terms of increased activity in midbrain dopamine neurons (Bavelier et al., 2010; Howard-Jones et al., 2011). Such theories have been based on the ability to predict correct responses from estimated reward system response in a learning game (Howard-Jones et al., 2011), from children’s preference for, and adult emotional response to, reward uncertainty (Howard-Jones and Demetriou, 2009), and from laboratory-based and classroom-based measures of improved motivation and learning in response to reward uncertainty (Ozcelik et al., 2013; Devonshire et al., 2014). However, to our knowledge, this is the first reported ventral striatal activation in relation to answering an educational question. Further research might valuably identify how the three characteristics of the Game-based condition employed here (competition, escalation and uncertainty) contributed to this response and to the apparent increase in learning in the Game-based condition, as well as clarify the relationship between these two outcomes.

Sorting and comparison of trials based on correctness of question response revealed increased ventral striatal activation bilaterally for positive feedback in both the Game-based condition and in the left VS for the Self-quizzing condition. Understanding of the brain’s reward circuitry has been established chiefly through its robust response to physical pleasures, and recent studies have shown that the same networks are activated in response to social rewards such as praise (Lieberman and Eisenberger, 2009). Being well regarded, treated fairly, cooperating with others and seeing competitors lose points have all been reported to activate the VS (Rilling et al., 2002; Moll et al., 2006; Tabibnia et al., 2008; Howard-Jones et al., 2010). The present study supports the notion that the responsiveness of the human reward system to social stimuli may extend to educational contexts. In the Game-based condition, the context might be described as strongly social, in the sense that participants were being observed by, and competing with, a peer. Indeed, this was done on the basis that peer presence might enhance reward system response, as observed in a study of teenage risk-based decision making (Chein et al., 2011). Less predictable, however, was the reward activation identified in the Self-quizzing condition. No competitors or peers were present in this condition (other than possibly the experimenter, who was monitoring the experiment in the control room). Participants knew their final score would be published sometime later with others and a certificate would be provided for the highest score, but it had also been emphasized that this would all be done anonymously. Therefore, our results suggest that simply answering a question, and also being provided with positive feedback, may themselves be rewarding experiences in an educational context, even without peers being present.

We have chiefly discussed improvements in learning observed in the Game-based condition in terms of reward system response, but it is important to point out that these effects might, at least in part, be explained in other ways. Alongside improvements in self-reported engagement, self-reported stress was also greater in the Self-quizzing condition compared with the Study-only condition, and increased further in the Game-based condition. All three defining characteristics of the Game-based condition (competition, escalation, and uncertainty) might conceivably have contributed to this stress. The stress would have been experienced in the context and close in time to the “to be remembered” material, and may have triggered hormones (corticosteroids, noradrenaline, corticotropin releasing hormone) suitable for enhancing memory (Joels et al., 2006). If the stress was related to impending judgement, then a desire to avoid humiliation could conceivably have contributed to greater attention to the learning task, and consequent deactivation of the default mode network.

## Conclusion

We have demonstrated links between deactivation of the DMN and educational learning. We believe our results support the proposed usefulness of the concepts and techniques of cognitive neuroscience in education, and particularly in regard to the design of technology-enhanced learning (Howard-Jones et al., 2014b). More specifically, the identification of neural markers associated with educational notions of engagement may facilitate new possibilities for “educational and neuroscience research efforts to inform one another in increasingly rapid cycles” (McCandliss, 2010, p. 8050).

## Conflict of Interest Statement

The authors declare that the research was conducted in the absence of any commercial or financial relationships that could be construed as a potential conflict of interest.
